# Une encéphalopathie postérieure réversible révélant une glomérulonéphrite post infectieuse

**Published:** 2012-12-18

**Authors:** Adnane Mohamed Berdai, Mustapha Harandou

**Affiliations:** 1 Service de réanimation Mère et enfant, centre hospitalier Hassan II, Fès, Maroc

**Keywords:** Encéphalopathie postérieure réversible, glomérulonéphrite, post infectieuse, œdème cérébrales, Glascow coma, posterior reversible encephalopathy, glomerulonephritis, post infectious, cerebral edema, Glascow coma scale

## Images en médecine

L'encéphalopathie postérieure réversible (PRES) est un syndrome clinico-radiologique, qui se manifeste par des céphalées, une confusion, des troubles visuels et des crises convulsives et sur le plan radiologique par des anomalies de la substance blanche suggérant un ‘dème des régions cérébrales postérieures pariéto-occipitales. C'est une complication rare d'une élévation brutale de la pression artérielle mais peut se voir dans d'autres circonstances, comme au décours d'un traitement immunosuppresseur. Son mécanisme est expliqué par une lésion de la barrière hemato encéphalique entrainant un ‘dème vasogénique réversible. La rareté de l'innervation sympathique des territoires cérébraux postérieurs explique sa localisation habituelle. Souvent décrites chez l'adulte, Nous rapportons le cas d'un enfant de 13 ans qui a présenté à la suite d'un épisode d'angine mal traité, une fièvre persistante, des douleurs abdominales diffuses et des céphalées intenses; compliqués par des crises convulsives tonico clonique généralisées cédant au Diazépam. L'examen clinique montrait un Glascow coma scale à 12 sans signe de focalisation, une tension artérielle à 260/110 mmHg et une protéinurie positive à la bandelette urinaire. Le bilan biologique révélait des anti streptolysine O (ASLO) positifs et une insuffisance rénale aigue. La tomodensitométrie cérébrale montrait des de lésions hypodenses sous corticales bilatérales mal limitées dans les territoires postérieurs en rapport avec le PRES. Le diagnostic d'une glomérulonéphrite aigue post streptococcique compliqué d'un PRES a été retenu. L'enfant a reçu la Nicardipine en perfusion continue, L'évolution est marquée par la normalisation des chiffres tensionnels et la reprise de la conscience sans séquelles.

**Figure 1:**
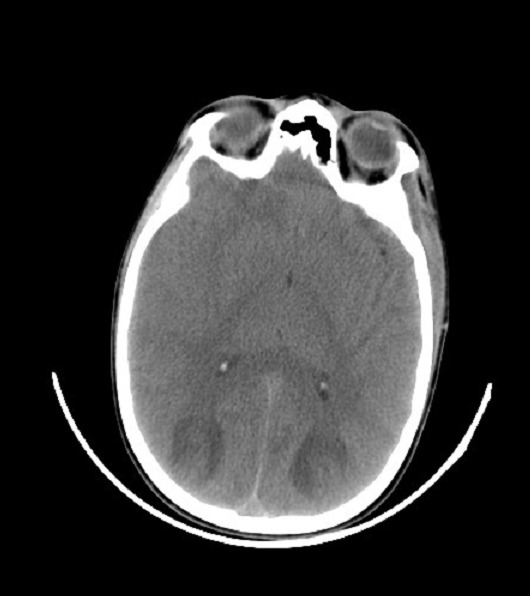
Coupe tomodensitométrique montrant des lésions hypodenses sous corticales bilatérales en regard des cornes occipitales

